# Cell Geometry Guides the Dynamic Targeting of Apoplastic GPI-Linked Lipid Transfer Protein to Cell Wall Elements and Cell Borders in *Arabidopsis thaliana*

**DOI:** 10.1371/journal.pone.0081215

**Published:** 2013-11-08

**Authors:** Chris Ambrose, Allan DeBono, Geoffrey Wasteneys

**Affiliations:** 1 Department of Botany, University of British Columbia, Vancouver, British Columbia, Canada; 2 Beaty Biodiversity Research Centre, University of British Columbia, Vancouver, British Columbia, Canada; Instituto de Biología Molecular y Celular de Plantas, Spain

## Abstract

During cellular morphogenesis, changes in cell shape and cell junction topology are fundamental to normal tissue and organ development. Here we show that apoplastic Glycophosphatidylinositol (GPI)-anchored Lipid Transfer Protein (LTPG) is excluded from cell junctions and flat wall regions, and passively accumulates around their borders in the epidermal cells of *Arabidopsis thaliana*. Beginning with intense accumulation beneath highly curved cell junction borders, this enrichment is gradually lost as cells become more bulbous during their differentiation. In fully mature epidermal cells, YFP-LTPG often shows a fibrous cellulose microfibril-like pattern within the bulging outer faces. Physical contact between a flat glass surface and bulbous cell surface induces rapid and reversible evacuation from contact sites and accumulation to the curved wall regions surrounding the contact borders. Thus, LTPG distribution is dynamic, responding to changes in cell shape and wall curvature during cell growth and differentiation. We hypothesize that this geometry-based mechanism guides wax-carrying LTPG to functional sites, where it may act to “seal” the vulnerable border surrounding cell-cell junctions and assist in cell wall fortification and cuticular wax deposition.

## Introduction

Epidermal cells demarcate the physical boundary between organism and environment. In aerial plant tissues, the epidermis serves to minimize water loss, restrict pathogen invasion, and facilitate organ growth. Starting as a uniform sheet of small boxy cells in young organs, epidermal cells expand in highly anisotropic patterns as the organ grows, creating a complex topology of epidermal cell shape and intercellular contacts. Anticlinal walls demarcate the areas of contact between epidermal cells (i.e. junctions), and are thus crucial components in epidermal functioning. Considering the epidermis as a structural composite akin to a brick wall, anticlinal wall junctions represent the mortar, and are thus structurally prone to mechanical stresses [1]. For this same reason, anticlinal wall junctions present the easiest route for an invading fungal hypha [2] and often separate and form holes in the leaf epidermises of *Arabidopsis* mutants with impaired cell adhesion [3]. The leaf epidermal cells of many species have wavy anticlinal walls, which are believed to enhance the structural integrity of the organ. Similar wavy boundaries have been observed in the nacre of seashells, where they assist in dissipating applied load forces over a larger area, thereby reducing the formation and propagation of cracks [4]. 

 In contrast to epidermal anticlinal wall junctions, the inner periclinal face of an epidermal cell is adjoined to the underlying cells of the ground tissues. This face may experience varying degrees of separation from the underlying cells, as in the case of the highly branched cells of the leaf spongy mesophyll, which gradually lose contact with their epidermal neighbors as they round up and expand to form air spaces [5]. Little is known of the mechanisms mediating this separation although in general, when cell wall thickenings are opposite one another between adjacent cells, the cells will separate between the thickenings [6]. This can be seen during stomatal pore formation [7], and during mesophyll cell differentiation [8,9]. Conversely, when wall thickenings alternate, as in leaf pavement epidermal cells, the cell junctions do not separate and the junctions become wavy as they expand [10,11].The border that surrounds every cell junction is particularly crucial both structurally and biochemically. The outer borders of epidermal cell junctions typically contain thicker cell walls, and are often fortified by the presence of extra cuticle [12,13]. Similar fortification is seen around junctions with and between cells of the ground tissue, such as collenchyma. The chemistry and mechanics of junction borders plays a role in determining intercellular adhesion, however, how their specific geometries regulates the distribution of apoplastic molecules is unknown. The chemistry and mechanics of junction borders play a role in determining intercellular adhesion [14] but how their specific geometries regulate the distribution of apoplastic molecules is unknown. 

 The current study was brought about by the observation that a glycophosphatidylinositol (GPI)-linked lipid transfer protein (LTPG) accumulates specifically at junctional borders. This junctional accumulation depends on the geometry of the border. As borders round up during cell expansion, the junction boarder accumulation of LTPG dissipates and the protein spreads out to the free region between cells. In mature epidermal cells, we often observed LTPG distributed in a pattern reminiscent of cellulose microfibrils. Using a YFP-tagged LTPG, we found that YFP-LTPG resides both in the apoplastic space between the plasma membrane and cell wall, as well as in the intercellular fluids prior to air filling. When we created artificial contact between a cell and coverslip, the YFP-LTPG fluorescence rapidly evacuated the region of contact and accumulated around its borders. Based on these observations, we propose that the varied YFP-LTPG accumulation patterns manifest from a simple mechanism, wherein YFP-LTPG distribution responds to geometrical changes within cell boundaries and becomes excluded from any sites of physical contact by a passive flow mechanism. 

## Results

### YFP-LTPG forms a striated pattern and accumulates over anticlinal walls

To observe the cellular distribution of LTPG, we imaged a fusion between citrine yellow fluorescent protein (YFP) and LTPG, driven by the native *LTPG* promoter in a complemented *ltpg-1* mutant background (_*pro*_ *LTPG:YFP-LTPG*) [15], and examined the distribution in multiple cell types using a confocal microscope. 

**Figure 1:**
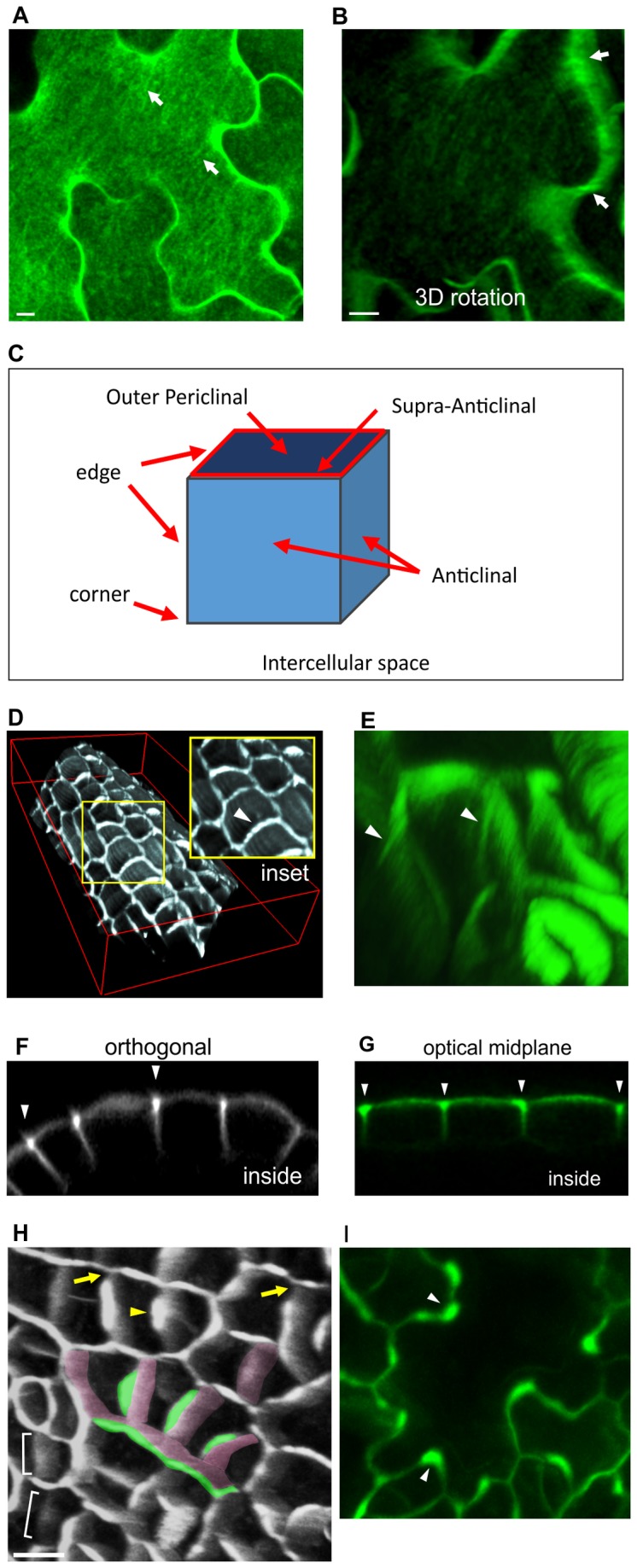
YFP-LTPG shows filamentous patterning and accumulation over anticlinal walls. **A** Mature cotyledon epidermal cell showing filamentous YFP-LTPG patterning (arrows). **B** 3D rotation of mature cotyledon epidermal cell shows filaments extending down anticlinal walls (arrows). **C** Schematic diagram showing nomenclature used in this article. **D** Tilted image from z-series 3D reconstruction showing anticlinal YFP-LTPG accumulation in unexpanded leaf petiole cells (arrowheads). **E** Radial striations (arrowheads) along anticlinal walls of unexpanded leaf petiole cells. Tilted image from 3D rotation. **F** Orthogonal slice from confocal Z-series illustrates YFP-LTPG fluorescence accumulation over anticlinal walls (arrowheads). **G** Optical midplane image of epidermal cells showing accumulation of YFP-LTPG over anticlinal walls (arrowheads). **H** Tilted image from 3D reconstruction of YFP-LTPG expressed in unexpanded leaf. Recently-formed cell walls (brackets) contain faint, homogeneous fluorescence. As the anticlinal wall matures, YFP-LTPG accumulates above it non-uniformly (green highlight), showing a gradual increase in fluorescence with increasing distance from three-way junctions (arrows). Green highlight shows outer edge enrichment; pink shows anticlinal walls. Arrowheads indicate example of outer enrichment site. Bottom panel shows orthogonal slice. **I** Mid-stage lobed leaf epidermal cell showing non-uniform accumulation of YFP-LTPG over anticlinal walls. Arrowheads indicate accumulation of YFP-LTPG within concave sides of cell lobes. Bars, 10 µm.

 In mature cotyledon and leaf epidermal cells, YFP-LTPG often showed a striated distribution pattern on the outer periclinal cell faces, resembling the pattern of cellulose microfibrils found in this cell type [6] (Figure 1A, B). Incubation in 20 µM of the cellulose synthesis inhibitor 2, 6-dichlorobenzene (DCB) for 2 hours was sufficient to abolish the striated YFP-LTPG pattern, suggesting that this pattern is dependent on cellulose microfibril formation (Figure S1). In the small, boxy cells of unexpanded young leaves/cotyledons, YFP-LTPG fluorescence outlined the outer periclinal faces, accumulating directly above anticlinal wall junctions in a caulk-like pattern (hereafter referred to as the supra-anticlinal domain; See schematic) (Figure 1C-H). On the outer periclinal face of these cells, fluorescence was typically diffuse, showing no filaments, with the exception of occasional fibers extending down the anticlinal faces underlying the supra-anticlinal domain (Figure 1D, E). Supra-anticlinal fluorescence is clearly shown with orthogonal sections through confocal Z-series (Figure 1F), as well as optical confocal sections through the organ midplane (Figure 1G). Accumulation at the supra-anticlinal domain was not observed with plasma membrane control markers GFP-PIP2a, FM4-64 dye; or with cell wall marker PI (Figure S2). 

 In terms of a developmental time course, newly-established post-cytokinetic anticlinal walls show relatively little YFP-LTPG fluorescence and lack supra-anticlinal YFP-LTPG accumulation (Figure 1H, brackets; See Figure S3 for full developmental series). As the wall develops, supra-anticlinal fluorescence gradually accumulates in the middle of the new wall, forecasting the site of future lobe formation. As the new wall expands and becomes wavy, supra-anticlinal YFP-LTPG fluorescence becomes enriched at the curved regions (Figure 1I). 

### Striated and Peripheral YFP-LTPG Populations Are Apoplastic

**Figure 2:**
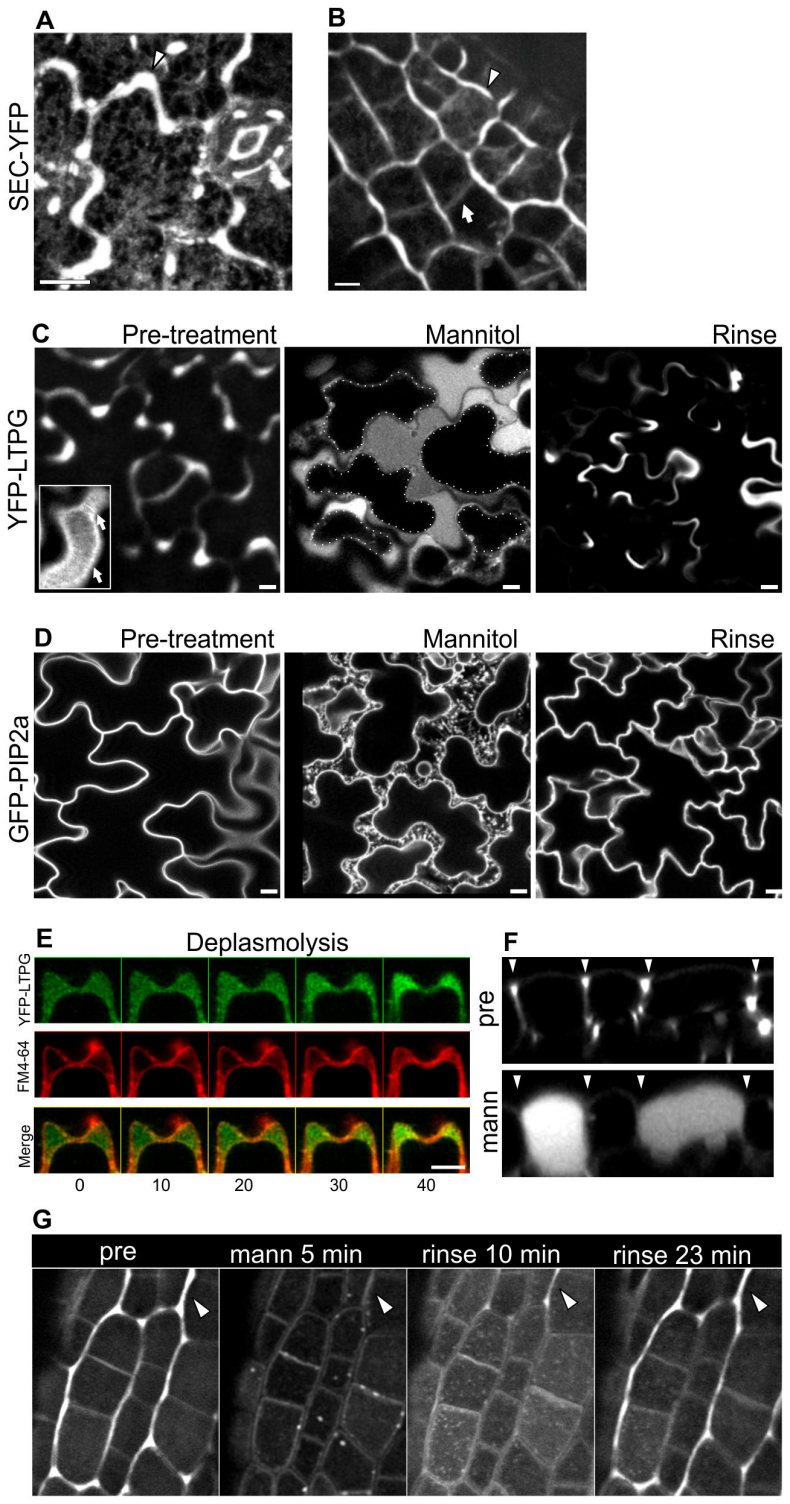
YFP-LTPG is apoplastic. A, B Apoplastic marker SEC-YFP accumulates over anticlinal walls, but shows no filamentous patterning. **A** Mature cotyledon epidermal cells (arrowheads show anticlinal enrichment). **B** Immature unexpanded cotyledon epidermal cells (arrowheads show anticlinal enrichment; arrow shows lack of enrichment at recently formed cell wall). **C** YFP-LTPG localization in leaf epidermal cells prior to treatment with mannitol, after 20 min in 500µM mannitol, and after 20 min rinsing in distilled water. YFP-LTPG fills in the apoplastic space between cell wall and plasma membrane in plasmolysed cells. Plasmolysis was complete in under 10 minutes, and rinsing in water restored initial pattern over anticlinal walls Inset shows striated YFP-LTPG fluorescence at the outer cell surface prior to plasmolysis. Arrows show striations. **D** GFP-PIP2a localization in leaf epidermal cells prior to treatment with mannitol, after 20 min in 500µM mannitol, and after 20 min rinsing in distilled water. GFP-PIP2a localizes to the retraced plasma membrane and accompanying Hechtian strands. YFP-LTPG does not localize to the plasma membrane, and becomes concentrated within the concave side of epidermal cell lobes as the protoplast expands during deplasmolysis. Co-staining of YFP-LTPG and FM4-64. **E** Loss of outer anticlinal enrichment and apoplastic filling of YFP-LTPG during plasmolysis. Arrowheads indicate anticlinal enrichment regions before and after plasmolysis. Shown are orthogonal views from cotyledon epidermal cells. **F** Plasmolysis results in rapid and reversible loss of outer anticlinal YFP-LTPG fluorescence (arrowheads). Bars, 10 µm.

The finding that intrinsic plasma membrane proteins are not co-distributed with LTPG suggested that YFP-LTPG might be accumulating in the apoplast. To test this, we engineered a secreted apoplastic marker consisting of an ER secretion signal sequence linked to YFP (sec-YFP). Like YFP-LTPG, sec-YFP was enriched in the supra-anticlinal domain (Figure 2A, B). Unlike YFP-LTPG, however, secYFP showed no detectable striated patterning. To confirm that the supra-anticlinal LTPG domain is indeed apoplastic, YFP-LTPG-expressing leaves were plasmolyzed by treatment with mannitol (500 mM, 10-15 minutes). Upon plasmolysis, both the striations and supra-anticlinal distributions were lost, and YFP-LTPG filled in the enlarged apoplastic space between the plasma membrane and wall (Figure 2C). In contrast, the control marker GFP-PIP2a labeled the plasma membrane and the Hechtian strands connecting to the wall during plasmolysis (Figure 2D). Fluorescence patterns were restored for YFP-LTPG and GFP-PIP2a upon deplasmolysis in water, typically within 5-15 minutes (Figure 2C, D). Time-lapse imaging of YFP-LTPG and FM4-64 together show the absence of YFP-LTPG in the plasma membrane, and deplasmolysis at a wall lobe shows that as the expanding protoplast fills up, YFP-LTPG fluorescence concentrates in the concave side of the lobe (Figure 2E).

Figure 2F shows that the plasmolysis-induced apoplastic YFP-LTPG filling is accompanied by loss of supra-anticlinal enrichment, suggesting that normally, the exclusion of LTPG from between adjacent epidermal cells is generated by turgor pressure. Indeed, rinsing plasmolysed cells in distilled water was sufficient to rapidly restore supra-anticlinal fluorescence (Figure 2G). Taken together, these observations show that both the striated and supra-anticlinal YFP-LTPG fluorescence distributions are apoplastic, and require sufficient turgor pressure to be maintained. 

### Cell-cell junction topology affects apoplastic YFP-LTPG distribution asymmetries

**Figure 3:**
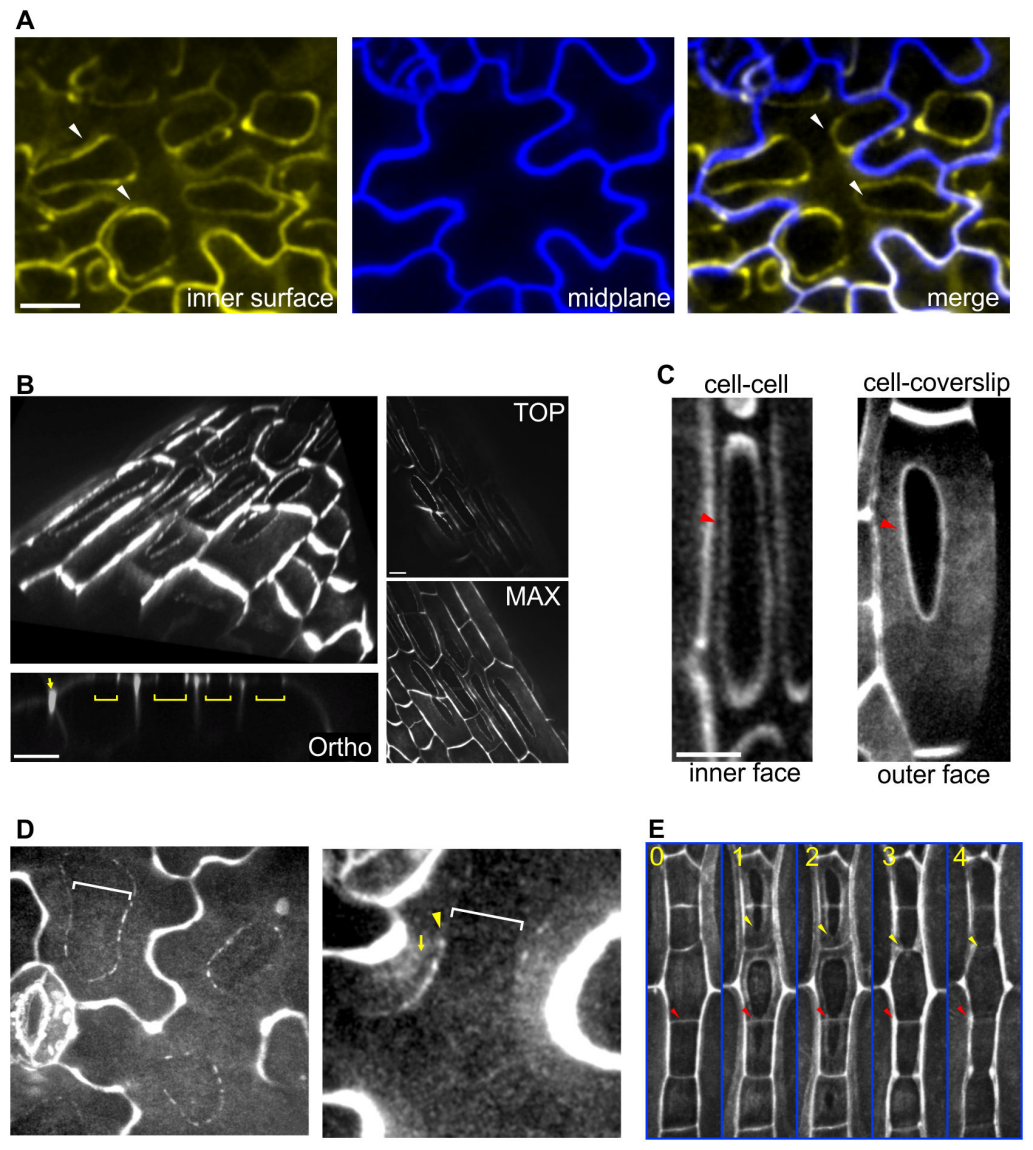
YFP-LTPG is excluded from cell-cell contacts and coverslip contact sites. **A** Cotyledon epidermal cells showing inner face (yellow) and midplane (blue), and overlay of these two planes. Arrowheads show ridges of fluorescence surrounding fluorescence free zones that are in contact with underlying mesophyll cells. **B** Contact with coverslip copies cell-cell contact clearing. 3D reconstruction tilted image from cotyledon petiole epidermal cells. Spaces occupy a plane on the outer periclinal face. Orthogonal views illustrate the planar nature of clearing (brackets), and show ridges as well. Arrow indicates normal anticlinal accumulation. Images in right panel show the top slice from the Z-series in C (top), and a maximum Z-projection of the series (bottom). **C** Clear zones at cell-cell (left panel) and cell-coverslip (right panel) contact sites. In both cases, ridges (arrowheads) surround clear zones. **D** Coverslip contact results in clear zones within the filamentous patterning (brackets) in mature epidermal cells. **E** YFP-LTPG contact clear zones form and enlarge as the coverslip is appressed over time. Images are ~1 minute apart. Yellow arrowheads track leading edge of tip clearing as it spreads to the lateral walls. Red arrowheads show where two clearing from adjacent cells meet and cross over their shared anticlinal wall. See also movie S1. Scale bars, 5 µm.

We next sought to investigate the asymmetric nature of YFP-LTPG distribution. We found that the distribution of YFP-LTPG varied depending on the topology of junctions between neighboring cells. In general, YFP-LTPG fluorescence is excluded from regions where cells contact one another (i.e. cell junctions), and accumulates around the borders of these junctions. This is easily demonstrated in cases where the internal periclinal face of epidermal cells partially contacts the underlying mesophyll/cortex cells, which are rounded and only in partial contact with the inner face of the epidermis (Figure 3A). In figure 3A, two focal planes from a confocal Z-series are pseudo-colored and overlaid, such that the relationship between the epidermal cells and the underlying mesophyll cells can be visualized. The regions that are in contact with the underlying mesophyll cells appear as darkened ‘holes’ with bright boarders surrounding them. In cases where the underlying cells are in full contact with the epidermis, such as those shown in Figures 1F-H, contact site fluorescence exclusion is complete on the internal periclinal faces, thereby creating a polarity in distribution to the outer cell faces.

 Since the supra-anticlinal fluorescence also surrounds cell-cell junctions, we hypothesized that applied contact on the outer epidermal face will also generate contact exclusion of YFP-LTPG. To test this, we created physical contact between the slide-mounted specimen and the glass coverslip by slowly wicking out the water between the slide and coverslip during imaging. Using this method, we observed distinct exclusion of fluorescence within regions where cells make contact with the coverslip (as assessed by the partial flattening of cells along a tangential plane) (Figure 3B). As with cell-cell junctions, YFP-LTPG fluorescence often accumulated around the boundary of coverslip contact zones (Figure 3C). In mature/fully expanded leaf epidermal cells, the striated fluorescence patterning was also excluded at sites of coverslip contact (Figure 3D). Contact exclusion was not observed in any form with the control markers GFP-PIP2 and FM4-64; and also not for cell walls labelled with propidium iodide (Figure S4).

 To observe the dynamics of contact clearing, we used 4D imaging while altering the degree of coverslip-cell contact over time. Figure 3E shows an example of this, wherein cell-coverslip contact is initiated and gradually increased over the time course, which lasts roughly 5 minutes (See also Movie S1). Contact clearings moved from the cell center (i.e. the highest point) to the periphery over time. Clearing dynamics were remarkably rapid and reversible, showing both clearing and filling within the highest temporal resolution we were able to achieve, which was typically 30-60 seconds between acquisitions. This directional opening/closing is consistent with a malleable convex surface gradually coming into contact with a hard flat surface. In the last time point of the series, the outer faces of several cells were flattened to the plane of the anticlinal edges. Here, the fluorescence was excluded from the entirety of the outer cell face, being thus relegated to the supra-anticlinal domain. 

 These data suggest that the absence of YFP-LTPG fluorescence at cell junctions is a manifestation of a more general contact exclusion mechanism. 

### Cell geometry affects apoplastic YFP-LTPG distribution asymmetries

**Figure 4:**
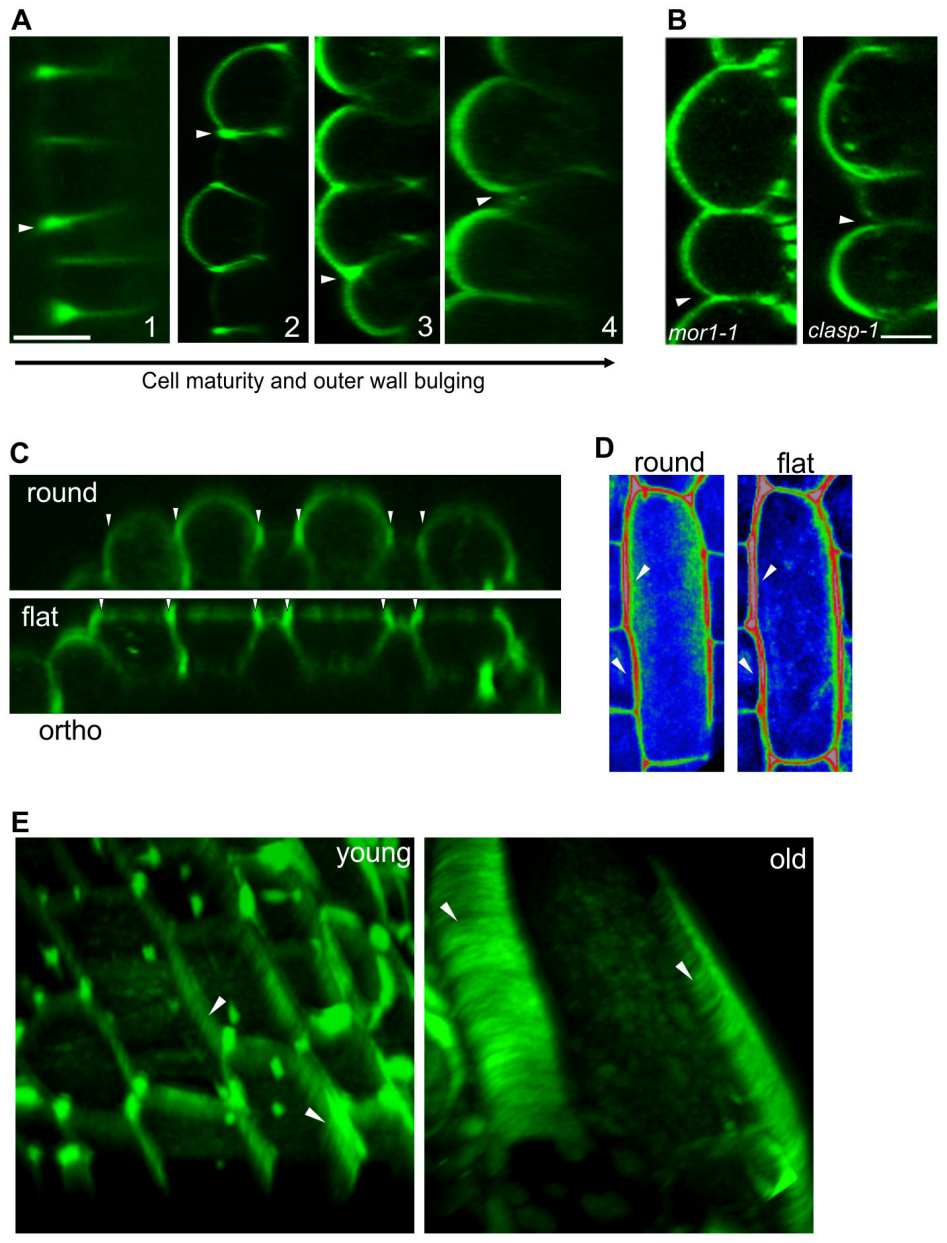
Cell geometry-dependent distribution of apoplastic YFP-LTPG. **A** Orthogonal views showing variable anticlinal and periclinal polarization. 1 - unexpanded leaf; 2 - young hypocotyl; 3 - mature hypocotyl; 4 - Loss of anticlinal enrichment in very bulged cells. **B** Loss of anticlinal enrichment in the bulbous cotyledon epidermal cells of *mor1-1* and *clasp-1* mutants. **C** Flattening of the outer periclinal face of bulging epidermal cells with coverslip generates increase in fluorescence over anticlinal walls (arrowheads). **D** Surface view showing increase in external anticlinal fluorescence (arrowhead) upon flattening of cell. **E** Formation of radial strations (arrowheads) along anticlinal walls in periclinally flattened epidermal cells. Left panel shows mid-stage cotyledon petiole cells, right panel shows mature hypocotyl cells. Bar, 5 µm.

The outer periclinal cell face of an epidermal cell is unique in that it is free from contact with other cells throughout their entire lifespan. Interestingly, we found that the degree of supra-anticlinal enrichment varies with the cellular geometry of the outer periclinal surface. Epidermal cells start small and cuboid, with flat external faces and sharp edges. As they mature, this external surface gradually bulges outward, and the cell edges become round [16]. We found that as the outer cell face bulges out, YFP-LTPG fluorescence extends across its entire surface, while the supra-anticlinal fluorescence decreases (Figure 4A). In cases of highly bulbous cells, the supra-anticlinal enrichment is strongly reduced or lost. This distributional change is exacerbated in mutants that have highly bulbous cells, such as *mor1-1* [17](after 48 hours at 29° C restrictive temperature) and *clasp-1* [18,19](Figure 4B). 

 The correlation between cell surface flatness and supra-anticlinal YFP-LTPG fluorescence lead us to hypothesize that the fluorescing borders surrounding contact-clear zones result from the flattening generated within contact zones. To test this, we asked whether complete flattening of the outer periclinal cell surface of bulgy cells can redistribute periclinal YFP-LTPG all the way to the anticlinal walls, thereby mimicking the distribution pattern in young, flat cells. Indeed, flattening the periclinal face in bulgy cells with a coverslip redistributed the periclinal fluorescence to the supra-anticlinal domain (Figure 4C, D). Notably, contact-induced redistribution to the periphery often forced the apoplastic YFP-LTPG down into the anticlinal walls, where it localized to filamentous striations (Figure 4E). 

### Filling of sub-epidermal intercellular spaces by YFP-LTPG

**Figure 5:**
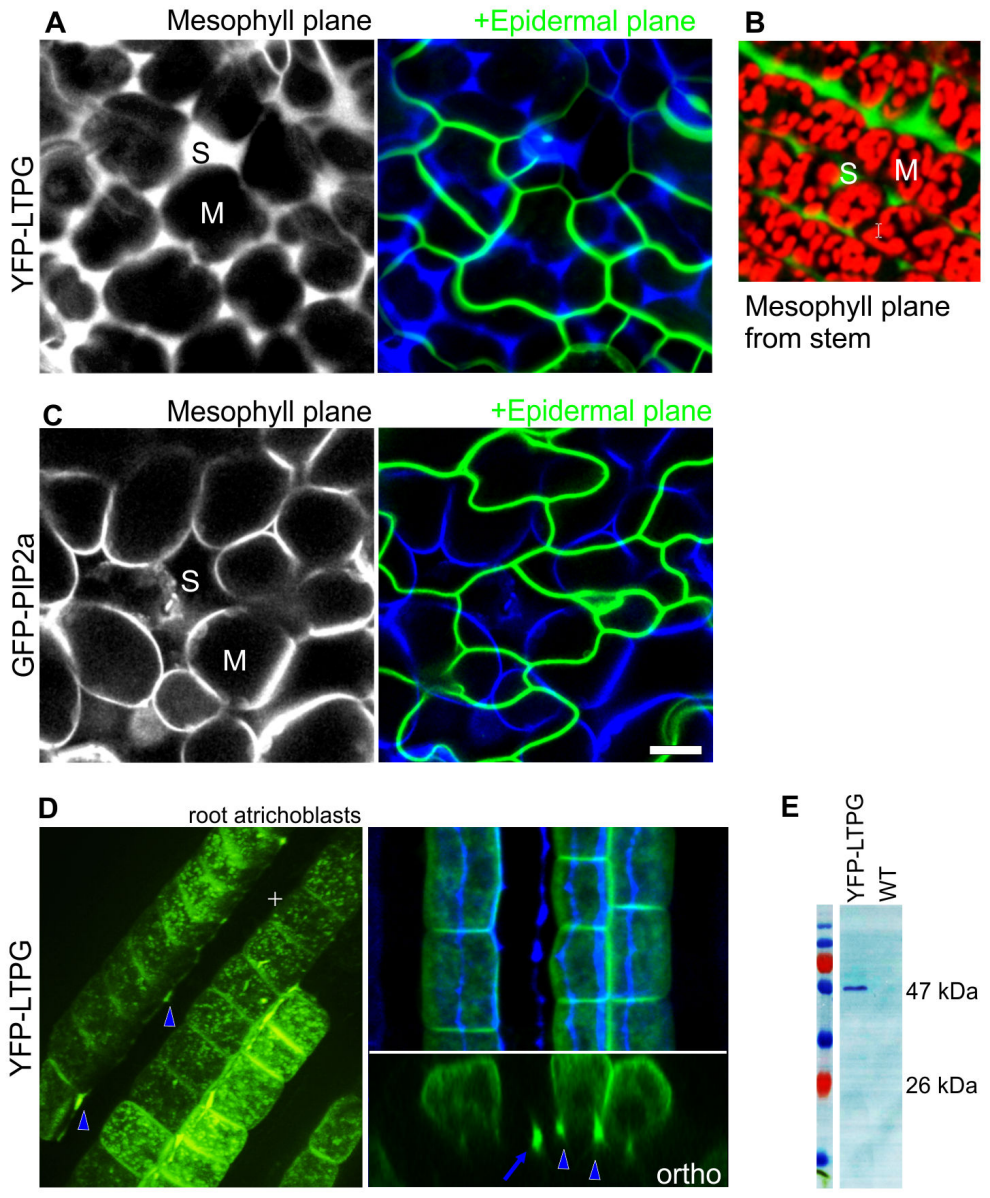
YFP-LTPG accumulates in intercellular spaces below epidermis. **A**-**C**. Intercellular localization of YFP-LTPG (A, B) and control plasma membrane marker GFP-PIP2A (C) in young cotyledon cells. B shows subepidermal intercellular fluorescence in a stem. Mesophyll cells visualized with chloroplast fluorescence (red). For A and C, the left panels show a single optical plane through the mesophyll layer below the epidermis. The blue/green panels show merged images with the inner slices pseudo-colored blue, and the optical midplane of the epidermal cells shown in green. M = mesophyll cell; S = intercellular space. **D** Intercellular YFP-LTPG distribution in roots. Fluorescence is limited specifically to atrichoblasts of the elongation zone (EZ). Left panel shows 3D reconstruction (arrowheads indicate subepidermal fluorescence lining inner borders of atrichoblasts). Right panel shows dual plane overlay, wherein subepidermal fluorescence appears as blue strips behind the green cell files in the overlay, and is indicated by arrowheads in orthogonal views. Arrow indicates subepidermal intercellular fluorescence beneath non-expressing cell file. **E** YFP-LTPG fluorescence is not due to free YFP. Western blot using anti-GFP shows full length YFP-LTPG band at 47 KDa. Bars, 5 µm.

In addition to its apoplastic accumulation patterns, YFP-LTPG often accumulated within the extracellular spaces below the epidermis (Figure 5A, B), in contrast to the plasma membrane marker GFP-PIP2a, which labeled only the plasma membrane (Figure 5C). The intercellular space filling was observed typically for young leaf cells, when the spaces still contain fluids prior to air filling [5]. Intercellular fluorescence was observed in roots as well, where the expression of YFP-LTPG was restricted to elongating cells within the atrichoblast (non-hair forming) cell files (Figure 5D). YFP-LTPG fluorescence often also appeared beneath non-expressing cells, indicating movement of the extracellular protein. Anti-YFP western blots showed the presence of full-length YFP-LTPG, excluding the possibility that the extracellular fluorescence is free YFP, cleaved from the YFP-LTPG (Figure 5E).

## Discussion

### Balloon in a box model

**Figure 6:**
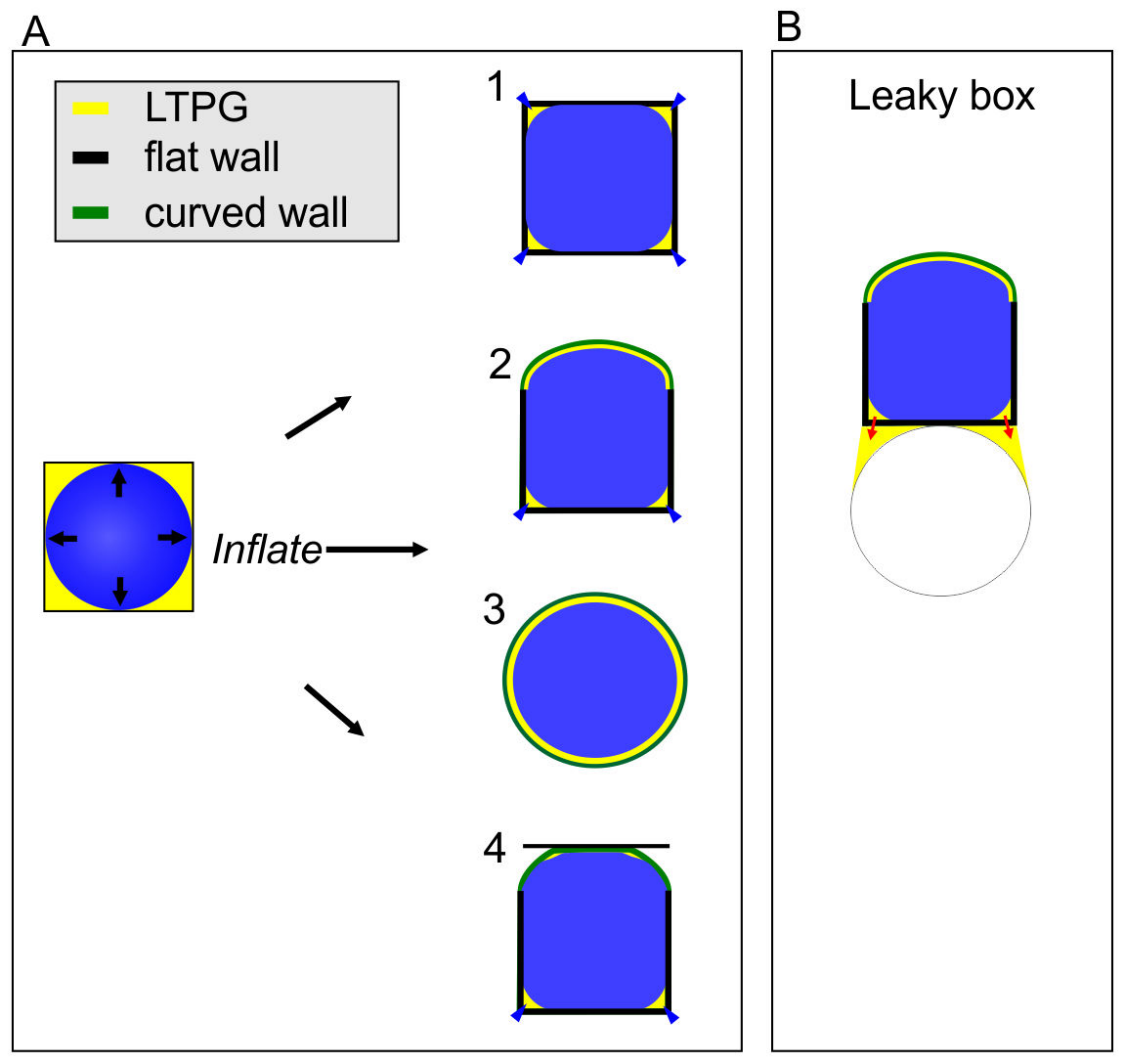
Hypothetical balloon in a box model to predict LTPG distribution patterns. **A** Three different scenarios for inflating a balloon in a box with differentially curved walls. As the balloon inflates, LTPG accumulates passively to spaces, which appear below sharply curved regions of the cell. The box 1 is analogous to a young boxy epidermal cell. Box 2 is analogous to a bulbous mature epidermal cells. Box 3 represents the hypothetical scenario of a perfectly round/walled sphere, which results in perfectly uniform LTPG distribution. Box 4 is analogous to our cover glass flattening scenario, wherein induced flattening of a rounded surface generates small regions of curvature bordering the flattened region. **B** Balloon in a leaky box model, wherein the naked internal walls of the epidermal cells created by the separation of underlying mesophyll cells leak apoplastic LTPG.

Based on our data, we propose a hypothetical model for YFP-LTPG distribution, which is nicely illustrated using the analogy of a balloon in a box (Figure 6). When the balloon (protoplast) inflates, it gradually comes into contact with the box walls (cell wall) and fills in the air spaces (analogous to liquid-filled apoplast *in planta*). As the expanding balloon comes into contact with the walls of a cube-shaped box, the last spaces to be filled by the balloon are the edges and corners. If a particular box wall is allowed to selectively yield to the pressure of the balloon by bulging out, the pressure between the balloon and wall gradually spreads out more uniformly over the entire face. At the same time, the edges bordering the bulging wall become more rounded and the protoplast then uniformly fills in those spaces. This predicts that variations in the uniformity of air spaces is generated ultimately by variations in wall curvature throughout the box, with perfect uniformity appearing only in a spherical structure. Factors secondary to the fundamental curvature factor include the volume occupied by the balloon, and the mechanical strength of the box. 

 Applying the balloon in a box model to epidermal cells accurately predicts the observed YFP-LTPG distribution patterns. As a cell grows and changes shape (i.e. changes wall curvature), the topology of cell junctions and junction borders surrounding it change as well. Regions of the wall that are free from contact with neighboring cells (such as the outer periclinal region) are able to bend out in response to the stress induced by the turgor pressure of the protoplast. Regions of walls that are in contact with other cells (such as anticlinal wall junctions) are unable to bulge in response to turgor stress due to the counter-pressure from the neighboring cell. Similarly, differentially thickened anticlinal walls, such as those in leaf pavement cells, will yield less to turgor stress at the thickened regions. These variations in wall yield-ability generate increasingly complex patterns of wall curvatures. When we flattened a bulbous (low curvature) cell face, YFP-LTPG accumulated within the newly formed regions of higher curvature bounding the contact site. As shown in Figure 3, this mimics the accumulation around epidermal-mesophyll junction regions. Fundamentally, apoplastic YFP-LTPG distribution patterns may result from local variations in wall curvature; accumulating passively to highly curved wall regions. 

 Biologically, passive accumulation of apoplastic YFP-LTPG beneath areas of high wall curvature may provide an efficient way to target and increase its concentration at its site of action. For example, the observed exclusion of protein from anticlinal epidermal walls passively enriches apoplastic lipid-bound LTPG along the top and bottom edges of the anticlinal walls, thus ‘sealing the cracks’ with lipidic compounds to prevent pathogen attack and non-stomatal water loss [20,21]. This is consistent with the accumulation of cuticle in these regions. When cell surfaces bulge out YFP-LTPG redistributes to the striated elements within this region, where it presumably would also provide lipidic compounds to those regions to fortify the wall there. This selective sealing of cell-cell junctions is reminiscent of the casparian strip in root endodermal cells, which restricts apoplastic water uptake into the root vasculature.

### Balloon in a Leaky Box?

How LTPG moves through the cell wall into the intercellular spaces is unclear. We speculate that pressure from the turgid epidermal protoplasts may be sufficient to squeeze the components out through the curved junctional borders of a porous primary cell wall, analogous to a balloon in a leaky box (Figure 6B). In this hypothetical model, the differential permeability of an anisoptropically expanding cell wall would allow for differential passage through the cell wall. For example, the wall within the supra-anticlinal region is known to be pectin-enriched and is thought to regulate wall porosity [22]. 

### LTPG trafficking

We show here that YFP-LTPG has a prominent apoplastic and intercellular distribution in leaves and hypocotyls. In inflorescence stem epidermal cells, YFP-LTPG has previously been shown to localize to the plasma membrane [15]. This apparent discrepancy can be explained by the fact that *LTPG* expression is restricted to the earliest stages of development in developing leaf and hypocotyl cells, whereas it continues until quite late in development in the epidermal cells of inflorescence stems. In young leaf and hypocotyl cells, YFP-LTPG has already trafficked to its final destination in the apoplast. In contrast, the continued production of YFP-LTPG in inflorescence stem epidermal cells generates prominent localization to cytoplasmic compartments and the plasma. Interestingly, supra-anticlinal and subepidermal distribution of YFP-LTPG is apparent in Figure 5 of the DeBono et al publication although neither was mentioned [15]. Similarly, Figure 5B in our study shows intercellular YFP-LTPG in stems. 

### Relationship to cell wall microfibrils

When responding to turgor pressure, the outer face experiences no counter-pressure from neighboring cells, and is thus free to bulge outwardly during its expansion and development. This is reflected in the organization and composition of the cellulose microfibrils in the outer wall, which can be relatively disorganized compared to the strongly aligned ones within the inner wall [23,24]. The tendency for YFP-LTPG to accumulate non-uniformly on the outer periclinal surface in distinct striation patterns suggests a preference for wall regions in which recently synthesized cellulose microfibrils are deposited. The rapid loss of this pattern after treatment with the cellulose synthesis inhibitor DCB supports the idea that LTPG is indeed distributed along pathways delineated by cellulose microfibrils and other tension-bearing wall materials. Initially concentrated in the highly curved regions of the supra-anticlinal domain in young leaf epidermal cells, the YFP-LTPG striation pattern develops as cells become more outwardly bulging, and the curvature within the supra-anticlinal region diminishes. The apparent association of LTPG with cellulose microfibrils could either be through confinement of LTPG to sites where tensile stress is lowest or through a chemical affinity of LTPG to microfibrillar components. The low tension-seeking mechanism seems more plausible since DCB treatment will specifically prevent the formation of new tension-bearing elements but will not remove preexisting cellulose microfibrils from the wall. Interestingly, secreted YFP, which of course is foreign to plant cell walls, did not accumulate in striation patterns on the periclinal surface, which indicates that the apparent association of YFP-LTPG is a functional attribute of LTPG and not of apoplastic proteins in general. 

### Possible functional relationship with other LTPs

The group of LTPs with GPI anchors has been classified as a subfamily of Non-Specific LTPs (NsLTPs). These subfamilies include sporopollenin, suberin and wax-related LTPGs; with LTPG1 belonging to the wax subgroup [25]. DeBono et al, 2009 showed LTPG functions in wax transport in stems [15]. Our findings here of *LTPG1* expression in additional organs suggest additional functions. For example, atrichoblastic cells have been shown to contain possible wax crystals on their surface [26], which would give an explanation for the presence of LTPG in these cells. The wider range of LTPG expression also raises the possibility that LTPG1 can work with other LTPGs. This claim is substantiated by recent work on LTPG2, which has been identified and characterized in two studies [15,27], and appears to have a partially overlapping role with LTPG1 [27].

## Methods

### Plant materials and growth conditions

*Arabidopsis thaliana* Columbia ecotype plants were grown in continuous light conditions on vertical agar plates containing either Hoagland’s or ½ MS medium. Young expanding cotyledon cells were imaged at 3-4 days. Leaves were imaged prior to opening. . Plants were transferred to soil when roots became 1-2 cm long. Hypocotyls were imaged 7-10 days after radicle emergence. Images of stems were acquired from the area within 1 cm of the floral apex unless indicated otherwise.

In order to select for plants expressing sec-YFP, each bearing Basta® resistance, plants were sprayed 23 mg L-1 Basta® in dH2O (Bayer Corporation) every two days after seedling emergence for two weeks. 

### Molecular biology and construct design

All oligonucleotides used in this study are listed in Table 1. The constructs sec-YFP is cloned in the vector pNOS (DeBono and Rieseberg, unpublished). The vector pNOS contains the nopaline synthase transcription terminator amplified from vector pMDC32 [28] with H7 and H8 and subcloned into the XbaI and SacI restriction sites of pGreenII0229 [29].

 To clone sec-YFP, the sequence corresponding to the functional LTPG promoter, amino terminal signal sequence and citrine-YFP was amplified from proLTPG:YFP-LTP [15] using P41 and P44. This amplicon contained a 5' EcoRI and 3' SpeI restriction sites and a stop codon. The P41-P44 amplicon was cloned into the EcoRI and XbaI restriction sites of pNOS. 

 All constructs were introduced into GV3101 harboring two helper plasmids, pMP90K and pSOUP plasmid [29]. Transformed clones were selected on Lysogeny Broth medium containing tetracycline, gentamycin (sigma-aldrich.com), and kanamycin (goldbio.com) at concentrations 10, 25, 50 μg mL-1, respectively. The presence of constructs in recovered *A. tumefaciens* clones was confirmed with restriction digestion analysis. Plants were transformed with the floral dip method [30].

### Immunoblotting

Plants were grown as above on ½ MS medium containing 1.2% bactoagar, 1% sucrose, at pH 5.9 and harvested at13 days after planting. For each genotype, 6 seedlings were ground in 200 µL of 2.5X Sample Buffer (0.156 M Tris-HCl, pH 8.0; 5% SDS; 12.5% Beta-Mercaptoethanol; 0.023% Bromophenol Blue; 3% Sigma Plant Cell Extract Protease Inhibitor Cocktail (#P-9599)) in microcentrifuge tubes at room temperature. We found that heating samples destroyed YFP-LTPG as previously reported for GPI-linked proteins [31]. Extracts were spun at 13,000 RPM for 10 minutes at room temperature. For SDS PAGE, 8 µL of supernatant was run at 80-120 V until fully separated. Next, proteins were transferred onto PVDF membrane (Biorad; 0.2 µm; #162-0177) at 15 V for 30 minutes in transfer buffer (25 mM Tris-HCl; 192 mM glycine, 10% methanol, pH 5.8). Following transfer, membranes were rinsed (3 times in TBST) and then incubated in blocking solution (3% BSA in TBST) for 1 hour at room temperature. Membranes were then incubated in mouse Anti-GFP (Roche #11814460001) at 1:2000 in BSA-TBST buffer for 3-4 hours at 4° C. After rinsing in TBST (3x 5 minutes) membranes were transferred to secondary antibody (goat Anti-Mouse HRP conjugate; Roche) at 1:5000 in 3% BSA/TBST buffer for 4 hours at 4°C, rinsed 3x 10 minutes each in TBST. Membranes were developed in HRP substrate developing solution (Sigma #T0565) for visualization. 

### Microscopy and image analysis

Images were acquired with three microscopes. The scanning confocal microscopes used were a Zeiss Pascal LSM5 (zeiss.com) and an Olympus Fluoview FV1000MPE (olympusfluoview.com). The third microscope was an UltraView VoX spinning disk microscope (perkinelmer.com). FM4-64 and propidium iodide stains were applied as described previously [15]. Images were processed using ImageJ software (http://rsb.info.nih.gov/ij) for linear contrast enhancements. Figures were assembled using either Corel Draw 16 (www.corel.com) or Inkscape 0.48 (www.inkscape.org) software. 

### Fluorescence Quantification

For quantification of polarity along the anticlinal walls, intensity profiles were generated from lines drawn along anticlinal walls from orthogonal views of confocal Z-series. Fluorescence intensity within large 3D confocal samples is known to attenuate with increasing depth and thus often requires correction [32,33]. In the shallow depths of the epidermal cells examined here however, little axial tissue-depth fluorescence attenuation was observed using controls (GFP-PIP2a, FM4-64, and PI), as can be seen in Figure S2. Moreover, orthogonal reconstructions produced images that were indistinguishable from optical midplane slices, which have no Z-axis decay, and are routinely used for showing protein polarities [34]. The small axial decay that we did observe with GFP-PIP2a was quantified and used as a standard to normalize YFP-LTPG measurements (Fig. S2). Ratios of outer to inner GFP-PIP2a anticlinal wall fluorescence intensities were taken from line profiles and averaged, resulting in an outer:inner ratio of 1.1, termed the decay coefficient (α). Similar decay rates were observed with FM4-64 and PI (See Fig. S2). Using α = 1.1, YFP-LTPG profile measurements were divided by 1.1 to obtain normalized measurements 

### Plasmolysis and coverslip experiments

For plasmolysis, cotyledons from 5-8 day seedlings were mounted onto flow cells, which consisted of two thin strips of vacuum grease at the middle edges running parallel to the slide length. Cotyledons or whole seedlings were mounted in water under a cover slip and imaged prior to addition of mannitol. Mannitol (500 mM) was then pipetted into the right side of the flow cell, while simultaneously wicking with filter paper on the left side to draw in the solution. Time-lapse imaging was then performed to observe the plasmolysis process. Rinsing in water was then performed in the same manner subsequent to plasmolysis. Following deplasmolysis, cells returned to pre-plasmolysis appearance, showing robust cytoplasmic streaming and normal fluorescence patterns, indicating our treatment regimens did not harm cell viability after deplasmolysis. Even partial plasmolysis (500 mM mannitol, 4 minutes) was sufficient to remove anticlinal YFP-LTPG fluorescence enrichment.

 For generation of coverslip contact with the specimen, the same flow cell method was used. As solution is slowly wicked out, the coverslip will gradually pull down toward the specimen. Contact with the specimen occurs well before any drying of the specimen, which remains submerged during the entire experiment. Flowing fresh solution in following flattening rapidly lifts the coverslip off the specimen.

## Supporting Information

Figure S1 **Treatment with DCB disrupts fibrillar pattern in contrast to DMSO controls.**
**A** YFP-LTPG control in 0.1% DMSO.
**B** YFP-LTPG in 20 µM DCB.
Bars, 5 µm.
(TIF)

Figure S2 **Control plasma membrane and wall markers lack accumulation over anticlinal walls.**
**A** YFP-LTPG is enriched over anticlinal walls, in contrast to uniform labelling of control GFP-PIP2a. Top panels show orthogonal slices of mature hypocotyl and cotyledon epidermal cells expressing GFP-PIP2a or YFP-LTPG. Graph in middle panel shows fluorescence intensity profiles along the anticlinal walls next to the dotted arrow. Several example traces of GFP-PIP2a and YFP-LTPG are shown. GFP-PIP2 fluorescence remains consistent with increasing depth, whereas YFP-LTPG decreases precipitously. Bottom panels are heat maps of YFP-LTPG and GFP-PIP2a fluorescence.
**B** Plasma membrane stain FM4-64 does not accumulate over anticlinal walls. Top panels show orthogonal slices of mature leaf epidermal cell containing YFP-LTPG and co-stained with 5 µM FM4-64. Intensity plots were drawn along the wall next to the dotted arrow. FM4-64 displays consistent fluorescence along the length of the anticlinal wall, while YFP-LTPG drops steeply with increasing depth. Bottom panels are heat maps of YFP-LTPG and GFP-PIP2a fluorescence.
**C** Cell wall stain propidium iodide does not accumulate over anticlinal walls. Top panels show orthogonal slices of mature leaf epidermal cell containing YFP-LTPG and costained with 10 µM propidium iodide.
**D** Intensity plots were drawn along the wall next to the dotted arrow. Propidium iodide displays consistent fluorescence along the length of the anticlinal wall, while YFP-LTPG drops steeply with increasing depth. Bars, 5 µm.
(TIF)

Figure S3 **Developmental sequence of YFP-LTPG distribution at anticlinal walls.** Brackets denote anticlinal wall of interest for each example.
(TIF)

Figure S4 **Control plasma membrane and cell wall markers do not show contact clearing.**
**A** Plasma membrane marker GFP-PIP2A does not clear from coverslip contact points. Maximum projection of Z-series and heat map pseudocolored image from orthogonal view of mature hypocotyl epidermal cells. Contact regions are indicated by brackets.
**B** FM-464 does not clear from coverslip contact sites. Brackets indicate coverslip contact regions. Orthogonal views on bottom show drop in YFP-LTPG intensity along contact region, but not FM4-64 intensity.
**C** Propidium iodide stain does not clear from coverslip contact points. YFP-LTPG in mature hypocotyl cells co-stained with propidium iodide. Brackets indicate clear zones. Arrowheads indicate lack of anticlinal accumulation in propidium iodide channel. Orthogonal views show drop in YFP-LTPG intensity along contact region, but not propidium iodide intensity. Bottom frame shows location of coverslip by increasing contrast (cs, arrow). Bars, 5 µm.
(TIF)

Movie S1 **Time series of showing initiation of coverslip contact with epidermal cells of cotyledon petiole.** Times are in minutes. Corresponds to Figure 3E, showing raw footage from which the montage in 3E was made.
(AVI)

Table S1 **Oligonucleotides used in this study.**
(DOCX)
