# Supplementary figures and images for: Cell Geometry Guides the Dynamic Targeting of Apoplastic GPI-Linked Lipid Transfer Protein to Cell Wall Elements and Cell Borders in Arabidopsis thaliana
Source: PLoS One. 2013 Nov 8;8(11):e81215. doi: 10.1371/journal.pone.0081215 (PMC3832655; doi:10.1371/journal.pone.0081215)

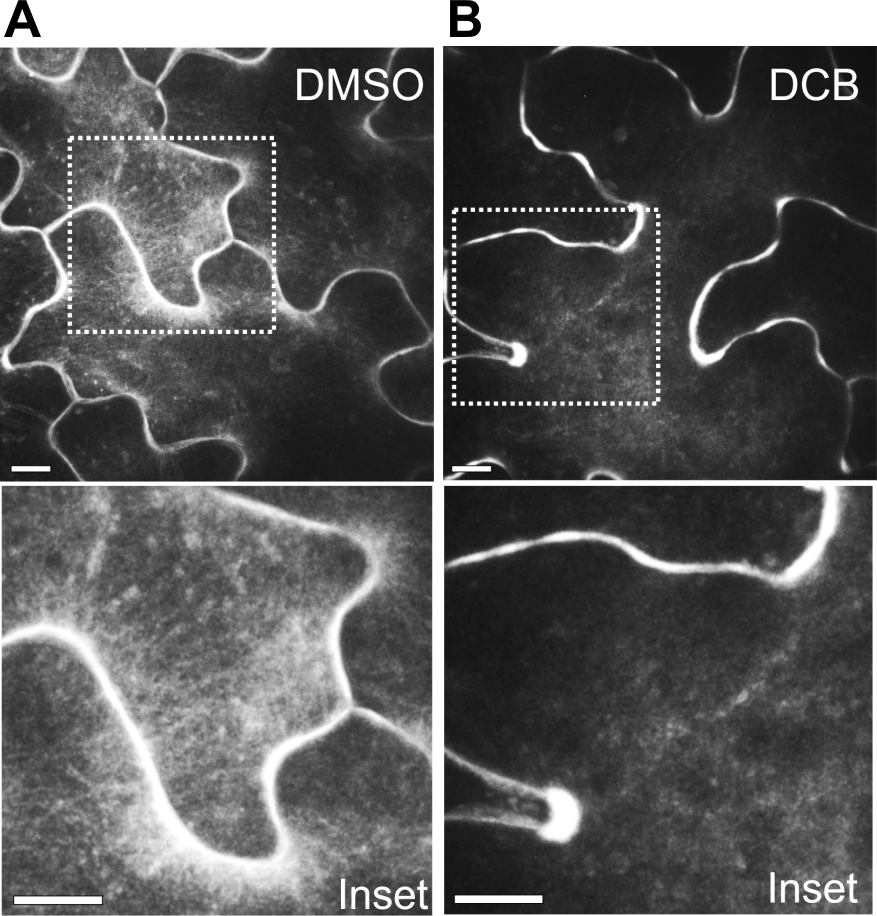

Supplement: Figure S1 — Treatment with DCB disrupts fibrillar pattern in contrast to DMSO controls. A YFP-LTPG control in 0.1% DMSO. B YFP-LTPG in 20 µM DCB. Bars, 5 µm. (TIF) [file pone.0081215.s001.tif]

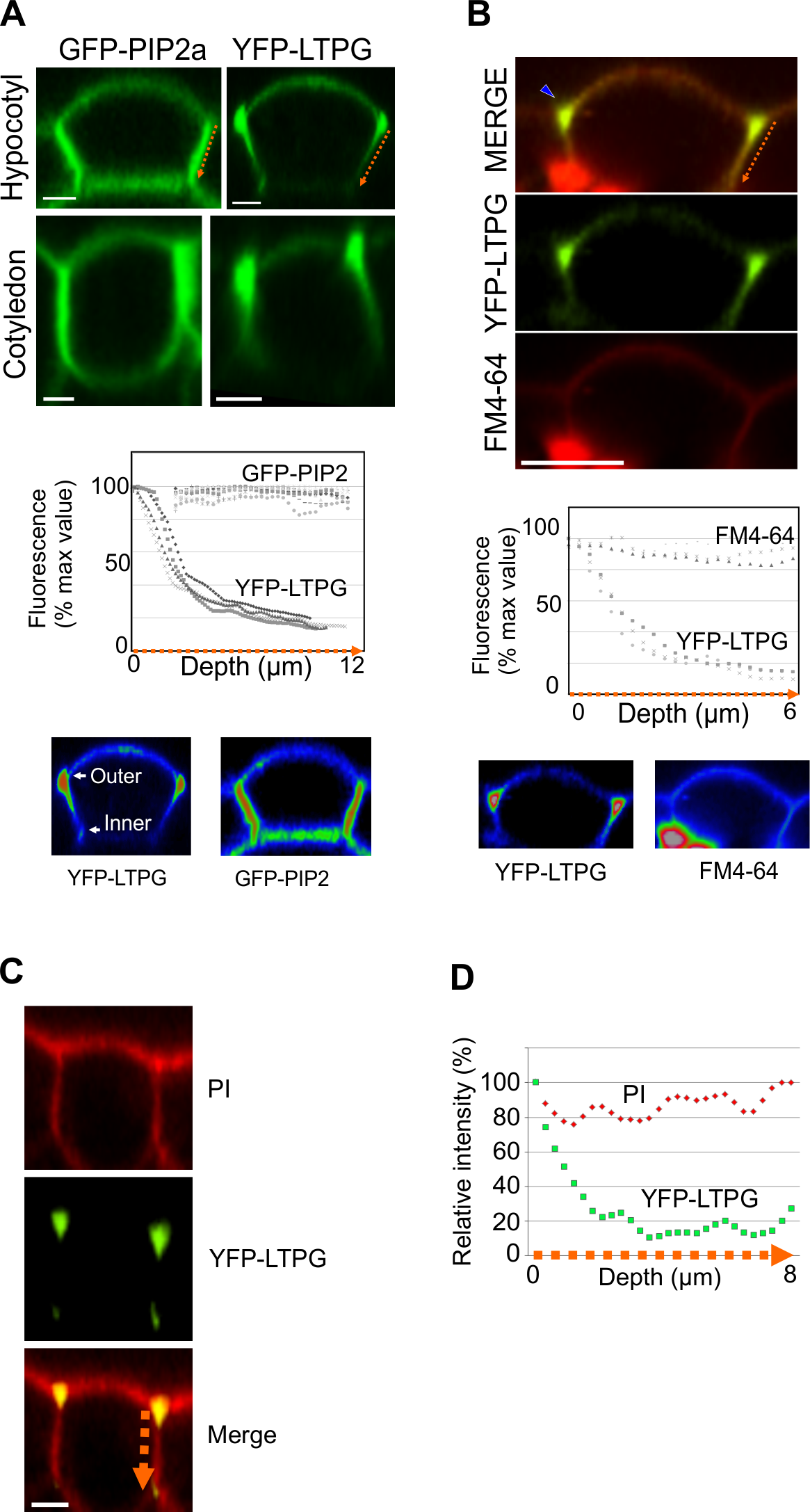

Supplement: Figure S2 — Control plasma membrane and wall markers lack accumulation over anticlinal walls. A YFP-LTPG is enriched over anticlinal walls, in contrast to uniform labelling of control GFP-PIP2a. Top panels show orthogonal slices of mature hypocotyl and cotyledon epidermal cells expressing GFP-PIP2a or YFP-LTPG. Graph in middle panel shows fluorescence intensity profiles along the anticlinal walls next to the dotted arrow. Several example traces of GFP-PIP2a and YFP-LTPG are shown. GFP-PIP2 fluorescence remains consistent with increasing depth, whereas YFP-LTPG decreases precipitously. Bottom panels are heat maps of YFP-LTPG and GFP-PIP2a fluorescence. B Plasma membrane stain FM4-64 does not accumulate over anticlinal walls. Top panels show orthogonal slices of mature leaf epidermal cell containing YFP-LTPG and co-stained with 5 µM FM4-64. Intensity plots were drawn along the wall next to the dotted arrow. FM4-64 displays consistent fluorescence along the length of the anticlinal wall, while YFP-LTPG drops steeply with increasing depth. Bottom panels are heat maps of YFP-LTPG and GFP-PIP2a fluorescence. C Cell wall stain propidium iodide does not accumulate over anticlinal walls. Top panels show orthogonal slices of mature leaf epidermal cell containing YFP-LTPG and costained with 10 µM propidium iodide. D Intensity plots were drawn along the wall next to the dotted arrow. Propidium iodide displays consistent fluorescence along the length of the anticlinal wall, while YFP-LTPG drops steeply with increasing depth. Bars, 5 µm. (TIF) [file pone.0081215.s002.tif]

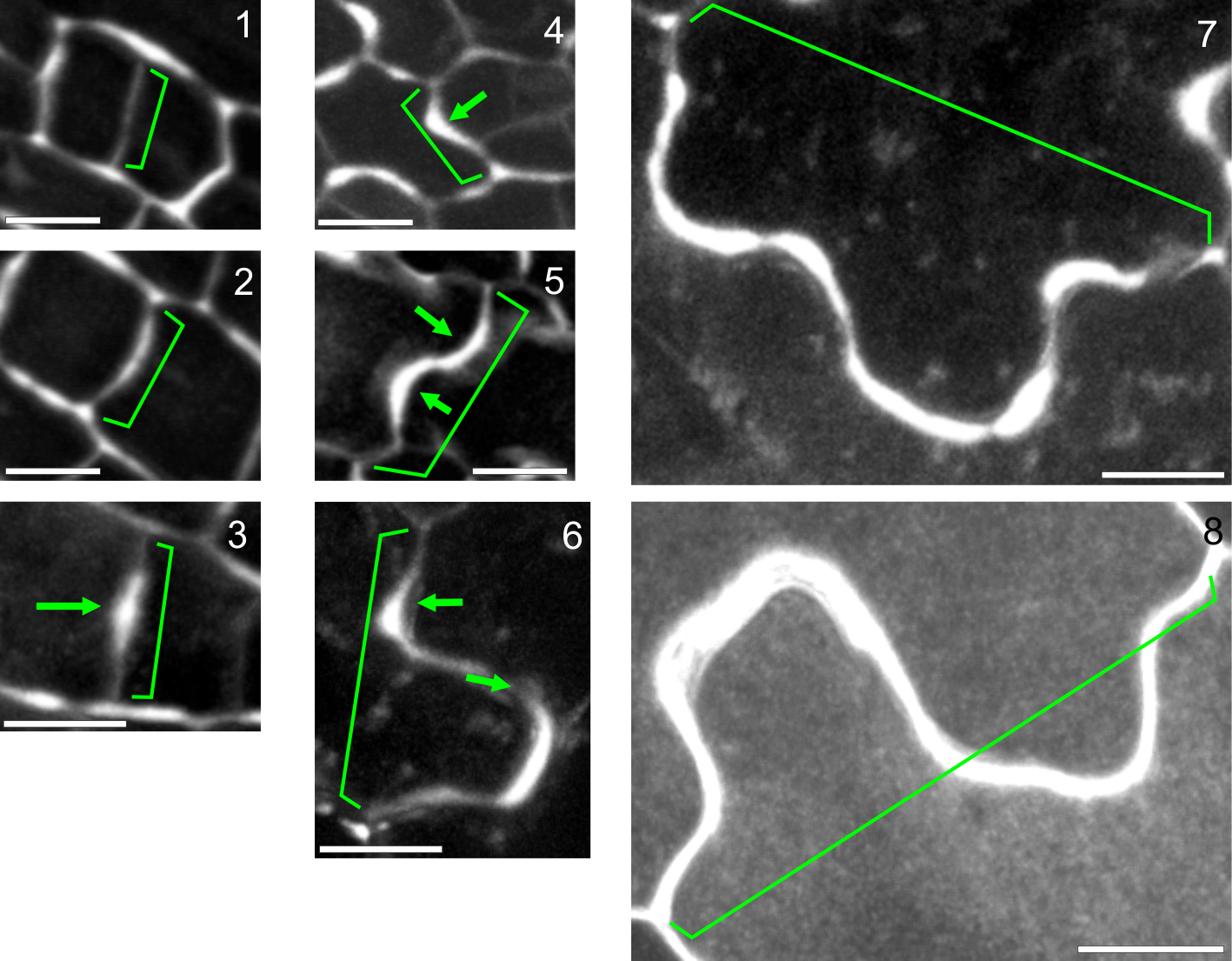

Supplement: Figure S3 — Developmental sequence of YFP-LTPG distribution at anticlinal walls. Brackets denote anticlinal wall of interest for each example. (TIF) [file pone.0081215.s003.tif]

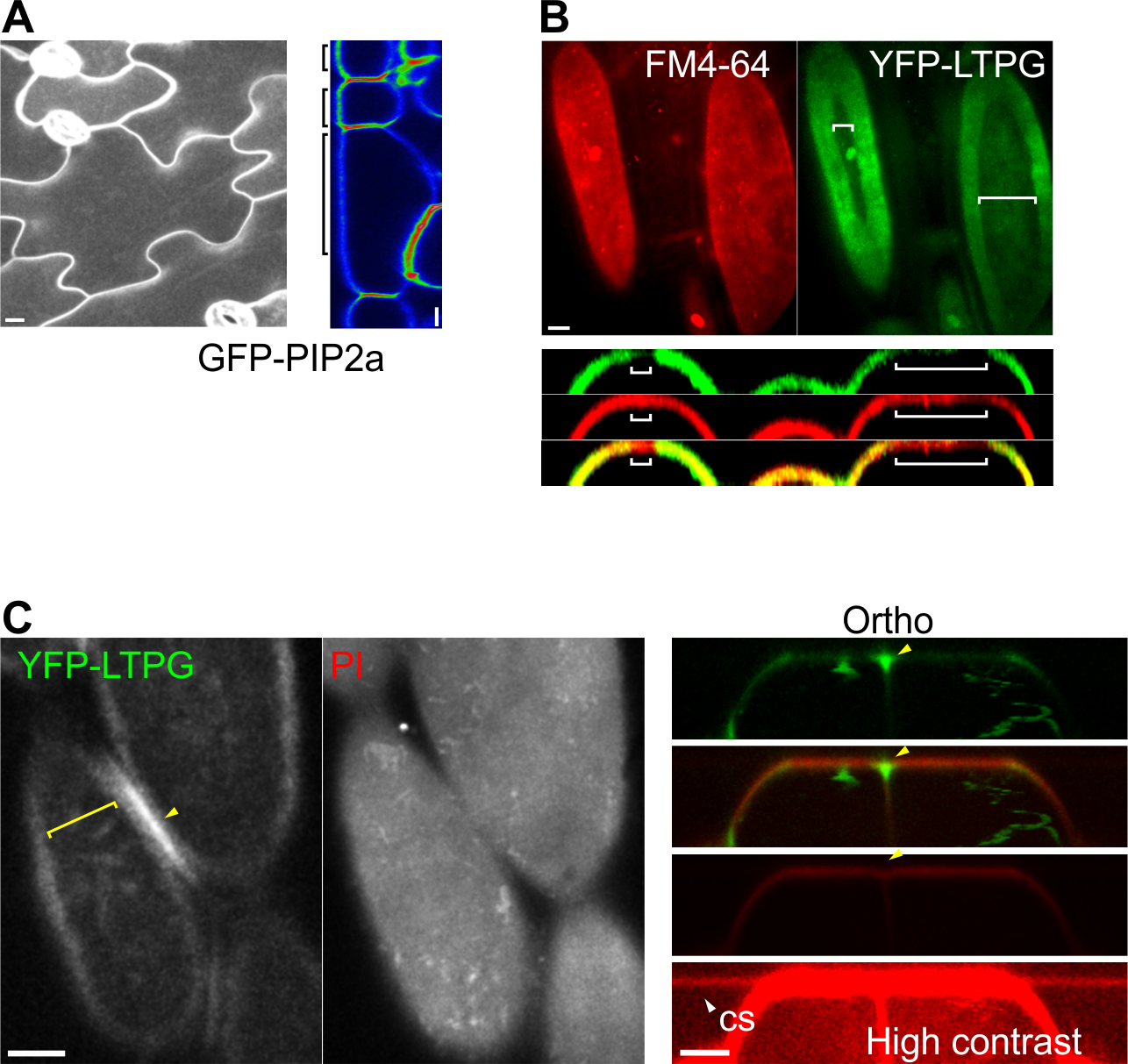

Supplement: Figure S4 — Control plasma membrane and cell wall markers do not show contact clearing. A Plasma membrane marker GFP-PIP2A does not clear from coverslip contact points. Maximum projection of Z-series and heat map pseudocolored image from orthogonal view of mature hypocotyl epidermal cells. Contact regions are indicated by brackets. B FM-464 does not clear from coverslip contact sites. Brackets indicate coverslip contact regions. Orthogonal views on bottom show drop in YFP-LTPG intensity along contact region, but not FM4-64 intensity. C Propidium iodide stain does not clear from coverslip contact points. YFP-LTPG in mature hypocotyl cells co-stained with propidium iodide. Brackets indicate clear zones. Arrowheads indicate lack of anticlinal accumulation in propidium iodide channel. Orthogonal views show drop in YFP-LTPG intensity along contact region, but not propidium iodide intensity. Bottom frame shows location of coverslip by increasing contrast (cs, arrow). Bars, 5 µm. (TIF) [file pone.0081215.s004.tif]
