# Supplementary material for: Cell Geometry Guides the Dynamic Targeting of Apoplastic GPI-Linked Lipid Transfer Protein to Cell Wall Elements and Cell Borders in Arabidopsis thaliana
Source: PLoS One. 2013 Nov 8;8(11):e81215. doi: 10.1371/journal.pone.0081215 (PMC3832655; doi:10.1371/journal.pone.0081215)
Supplement: Table S1 — Oligonucleotides used in this study. (DOCX) [file pone.0081215.s006.docx]

## Supplemental Table 1: Oligonucleotides used in this study.

***P** indicates 5’ phosphorylation. Restriction sites are underlined

| P4 | CGAGGTACCCGAGGTGGGACTGGAAAAAGGT |
| --- | --- |
| P7 | CCGTATACACTGCAGGGACTGGAAAAAGGTATAGAG |
| H7 | CATTGCAAGTCTAGAgaatttccccgatcgttcaaacatttggcaataa |
| H8 | CATTGCAAGGAGCTCagtaacatagatgacaccgcgcgcgata |
| P35 | cttgtcaaaTCTAGAcCCGGCAACTCCAGCTACGTC |
| P37 | ***P**tatcagtttttctttcttttgttcaattactcttttgcttgttgaagatcttgtttg |
| P41 | gcaatttaaGAATTCcgtcaaagacaaagcgcaaagacgaaaattcc |
| P44 | ctaacgtcgACTAGTatcAGGCGCAGCAGCACCAGCAGGATC |
| P45 | ***P**ATGGTTCCTGGAGGTGGAGGTGGAGCTGTG |
